# Caffeine Use in Huntington’s Disease: A Single Center Survey

**DOI:** 10.5334/tohm.945

**Published:** 2024-10-18

**Authors:** Jennifer Adrissi, Sarah Brooker, Alyssa Mcbride, Danielle Larson, Eric Gausche, Danny Bega

**Affiliations:** 1 University of California Los Angeles David Geffen School of Medicine, US; 2 Northwestern University Feinberg School of Medicine, US

**Keywords:** Huntington’s disease, Caffeine, Chorea

## Abstract

**Background:**

Anecdotal evidence suggests paradoxical caffeine overuse in individuals with Huntington’s disease (HD). A small retrospective study associated caffeine intake over 190 grams daily to earlier onset of HD symptoms. However, specific data on consumption habits is limited. This study aims to gather pilot data on caffeine use in people with HD, exploring motivations and consequences.

**Methods:**

Thirty adults with HD completed a survey on daily caffeine intake, its impact on symptoms, and consumption motivations through multiple-choice and open-ended questions. Descriptive statistics were used to analyze findings and compare them to general population data.

**Results:**

Caffeine intake ranged from 0 to 1400.4 mg/day, with a median of 273.2 mg/day and a mean of 382.5 mg/day. Seventy percent of participants with HD consumed more caffeine than the average for their age group in the general population. Additionally, 20% of participants and 38% of family members believed caffeine influenced HD symptoms, primarily anxiety.

**Discussion:**

People with HD typically consume more caffeine than the general U.S. population. Contrary to the hypothesis, higher caffeine intake was not associated with significant subjective worsening of HD symptoms. Further research with objective measures and multiple HD centers is necessary to guide screening and counseling on caffeine use in this population.

**Highlights:**

Participants with Huntington’s disease (HD) had increased caffeine intake compared to the general population, supporting previous anecdotal observations. Anxiety was the most affected HD symptom. Further research using objective measures of symptom burden and including multiple HD centers can help inform screening and counseling regarding caffeine use in this population.

## Introduction

Huntington’s disease (HD) is a neurodegenerative disorder which causes progressive neuropsychiatric decline and disability with features including involuntary movements, psychiatric symptoms, and cognitive impairment. Caffeine acts as an adenosine receptor antagonist and at higher doses can also act as a ryanodine receptor agonist and phosphodiesterase inhibitor [1]. Adenosine receptors have been implicated in the pathogenesis of multiple neurodegenerative disorders, including Parkinson’s disease (PD) and Alzheimer’s disease (AD) [1]. A polymorphism in the *ADORA2 A* gene, which encodes the adenosine 2 A receptor (A_2 A_R), has been shown to be associated with earlier age at onset of HD symptoms [2,3], suggesting that alterations in A_2 A_R expression or activity could impact HD phenotypes.

The role of adenosine receptors and caffeine in mouse models have had mixed findings, and there are limited human studies. In an HD mouse model, knockout of A_2 A_R was shown to significantly worsen motor symptoms and survival [4], and treatment with a selective A_2 A_R agonist delayed the progression of motor phenotypes in transgenic HD mouse models and ameliorated huntingtin protein aggregation [5,6]. In contrast, other HD animal models have shown possible neuroprotective effects of caffeine [7,8].

To date, there have been very limited analyses of caffeine intake in HD patients. One retrospective analysis of patients with HD in France demonstrated that caffeine consumption of greater than 190 grams per day was associated with earlier age of onset of HD symptoms [9]. Anecdotally, clinicians have noticed increased amounts of caffeine consumption in the HD population. This can seem paradoxical as the disease itself makes people irritable and hyperactive, and caffeine may exacerbate this. This study aimed to gather pilot data from a single-site HD clinic to compare caffeine consumption in HD with that reported in the general population and identify motivators for caffeine use and its subjective effects on HD symptoms. It was hypothesized that the participants with HD would have increased caffeine consumption compared to the general population with a subjective worsening of hyperkinetic symptoms such as chorea reported by participants and accompanying family members.

## Methods

This study was approved by the Institutional Review Board at Northwestern University. All adult patients seen at Northwestern University HD Clinic between April and November 2022 were approached about participating in the optional research survey, either at appointment check-in or check-out. Eligible participants had to have a genetically confirmed HD diagnosis, be over the age of 18, and have the capacity to consent to research participation. After obtaining informed consent, research staff verbally administered the paper survey after providing the printed document to each participant. The surveys asked about daily caffeine intake, impact on HD symptoms, and motivations for caffeine consumption using a multiple choice (checked boxes or circling options) and short free response questionnaire, with input from accompanying family members for a subset of the questions [Appendix A].

Surveys also collected demographics and general information about HD clinical course including (1) the presence of motor and psychiatric symptoms and years since symptom onset, (2) current HD medication use, and (3) Total Functional Capacity (TFC) score [10] – a scale ranging 0–13 used to evaluate patients’ capacity to work, manage activities and instrumental activities of daily living, and live independently. The TFC scores were used to assign HD stage of disease, with higher TFC scores indicating higher functioning and corresponding to a lower, less severe HD stage. TFC scores from 11–13 indicate stage I; 7–10 = stage II; 3–6 = stage III; 1–2 = stage IV; and score of 0 = stage V. No identifying participant information was collected.

### Statistical Method

Descriptive statistics were used to report survey findings and compare the sample’s average caffeine consumption to that of the general U.S. population by age using a publicly available summary of a nationally representative database of 37,602 individuals who completed the 2010–2011 Kantar Beverages Consumption Panel [11,12]. Estimations of average caffeine content (mg/fluid ounce) per type of caffeinated beverage were based on a reference by Mitchell, et. al., which combined information from sources including the USDA National Nutrient Database for Dietary Studies, the USDA National Nutrient Database for Standard Reference, the Nutrition Data System for Research, and popular product brand websites [13].

The Wilcoxon Rank Sum Test was used to assess for differences in caffeine use between sexes. A general linear model was used to evaluate influence on caffeine intake, controlling for age group, sex, and presence of motor and psychiatric symptoms. Spearman’s Rho calculations were performed to assess for correlation between years since motor or psychiatric symptom onset and daily caffeine intake.

## Results

Thirty participants with HD were recruited and completed the survey. A summary of participant demographics and median caffeine intake by age group are presented in Table 1. The median age range was 35–49, and there was a relatively even distribution by sex with 53% and 47% of the participants identifying as male and female, respectively. 27 of the 30 participants identified as White, and one participant identified as Hispanic/Latino. Most of the participants were in stage 1 (n = 12) or 2 (n = 13) of the disease. When combining all age groups, the median total caffeine intake per day was 273.2 mg/day, ranging from 0 to 1,400.4 mg/day. Twenty-one participants (70%) had an average daily caffeine consumption that was above the national average for their age. There was no significant difference between daily caffeine consumption between males and females (p = 0.73). When assessing the impact of age group, sex, and motor and psychiatric symptoms on caffeine intake using a general linear model, only the presence of psychiatric symptoms was associated with increased caffeine use, when controlling for the other factors (p = 0.03). There was no association between the years since motor or psychiatric symptom onset and caffeine intake (r_s_ = –0.02, p = 0.92; r_s_ = 0.21, p = 0.33, respectively).

**Table 1:** Sample Demographics and Caffeine Consumption.


AGE GROUP (yrs)	SAMPLE SIZE (n)	%N	MEDIAN TFC SCORE (range)	MEDIAN YEARS SINCE SYMPTOM ONSET (range)		MEDIAN CAFFEINE INTAKE (mg/day), (range)				NATIONAL AVERAGE – CAFFEINE INTAKE (mg/day)^11^

				Motor	Psych	Coffee	Tea	Soda	Total	Total

25–34	3	100%	13 (5–13)	7.5 (5–10)	3 (1–5)	190.4 (164.3–238)	0 (0)	0 (0–158.4)	190.4 (164.3–396.4)	137.3

35–49	13	100%	7 (0–13)	6 (0–18)	5 (2–14)	142.8 (0–1142.4)	50.6 (0–188.8)	70.4 (0–686.4)	260.8 (62.9–1400.4)	199.1

50–64	11	91%	12 (7–13)	3 (1–7)	4 (0–22)	95.2 (0–428.4)	70.8 (47.2–94.4)	0 (0–528)	237.2 (0–528)	225.5

65+	3	100%	8 (7–8)	5 (5–6)	5.5 (1–10)	285.6 (285.6)	0 (0)	52.8 (15.3–105.6)	338.4 (300.9–391.2)	207.3

All ages	30	97%	8 (0–13)	5 (0–18)	5 (0–22)	177.3 (0–1142.4)	59 (0–686.4)	20.9 (0–686.4)	273.2 (0–1400.4)	105.4*


%N = percentage of age group sample that are caffeine consumers. National average – Caffeine Intake (mg/day) = the average total caffeine (mg) consumed per person per day in the United States based on a national database of 37,602 individuals who completed the 2010–2011 Kantar Beverages Consumption Panel^11^. *Reference average is for age range 2–65+.

Further analysis of caffeine use by HD stage was not performed given the limited variability in HD stages within the sample. 27% percent of participants felt that they should cut down on caffeine intake, and 40% have been told by family and/or friends that they need to decrease caffeine consumption. Of those with a family member present (n = 21), 70% had been told to cut down caffeine use by that family member.

**Figure 1:**
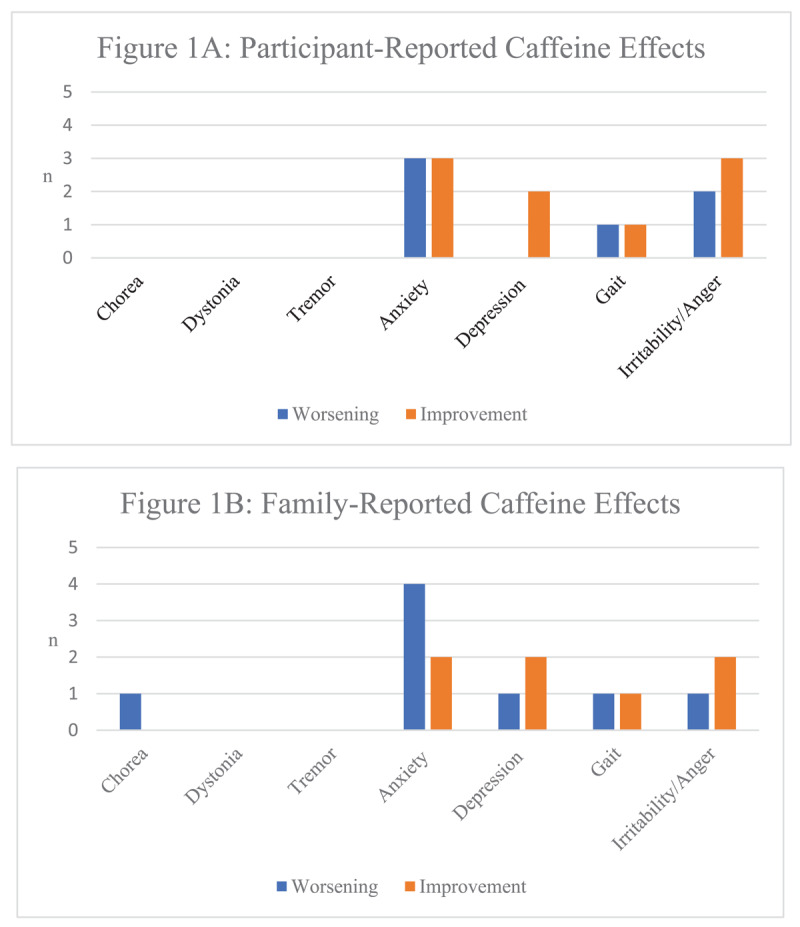
Reported Caffeine Effects on HD Symptoms.

Six of the 30 participants (20%) and eight of the 21 accompanying family members (38%) responded that caffeine affected HD symptoms. For HD patients the most affected symptoms reported were anxiety (10% improved, 10% worsened) and irritability (7% improved, 10% worsened) (Figure 1). Amongst accompanying family members, the symptoms that were most reported to be affected by caffeine were anxiety (10% improved, 19% worsened), depressive symptoms (10% improved, 5% worsened), and irritability (10% improved, 7% worsened). Notably, no HD participants and only one family member (5%) felt that caffeine worsened chorea.

Participants with HD were given nine statements regarding motivations behind caffeine use with Likert-based response options. Of the statements provided, they agreed or strongly agreed most with (1) “I like the taste” – 90%, (2) “It gives me extra energy” – 70%, and (3) “It makes me feel less sleepy” – 63%. Participants were also given the opportunity to add additional motivations via free response. The most common additional motivation provided was caffeine’s function as a “social ritual” or “habit,” mentioned by four of the 12 participants (33%) who provided free response input.

## Discussion

Each age group’s median caffeine intake per day exceeded the reported national average by age. This supports anecdotal patterns observed by HD clinicians in this population. Participants who reported psychiatric symptoms had significantly higher caffeine intake, but there was no clear correlation between years since symptom onset and average daily caffeine consumption. This analysis was limited by small sample size and some outliers. Reported motivations for caffeine use were comparable to the general population [14,15]. Given the presence of obsessive-compulsive symptoms and perseverative behaviors in HD, we consider this influence on the reported motivations of “ritual” and “habit” on the participants’ caffeine intake [16,17].

It was hypothesized that participants and their family members would report worsening of HD symptoms with caffeine use, but responses were more mixed than expected. Only 20% of participants with HD, compared to 38% of family members, reported that caffeine affected HD symptoms with some reporting symptom worsening and others symptom improvement. The discrepancy between the HD participant and family member responses could be, at least partially, attributed to anosognosia which is well-documented in the disease [18]. Interestingly, none of the participants with HD and only one of the family members noted worsening in chorea with caffeine use. Limitations of this study include the reliance on subjective reporting, potential for recall bias, and lack of a comparison control group of adults without HD. Another study limitation includes the lack of detailed evaluation of sleep symptoms as a contributor to and effect of caffeine use. Future studies investigating the differences in caffeine effects between caffeine sources (coffee, tea, soda, etc.) and including assessments of additional non-motor HD symptoms such as sleep, urinary, and gastrointestinal symptoms will add additional understanding of the motivations and effects of caffeine use in this group. Incorporating standardized and objective measures such as the Unified Huntington’s Disease Rating Scale and the Hamilton Anxiety Rating Scale could be helpful in future evaluations of caffeine’s effect on HD symptoms [19,20].

This is a pilot study that will hopefully inform similar, larger scale studies. While there are current investigations into disease-modifying therapies, HD management is currently supportive, and patients and their families rely on their physicians to counsel on therapeutics and lifestyle adjustments that reduce symptom burden and improve quality of life. Further research in this area is needed to inform caffeine recommendations for HD patients. Larger studies would allow for analysis of potential correlations between caffeine use and HD stage, time since symptom onset, and objective measures of disease burden.

## Data Accessibility Statement

The data used during the current study are available from the corresponding author on reasonable request.

## Additional File

The additional file for this article can be found as follows:

10.5334/tohm.945.s1 Appendix A. Study Survey.
