# Supplementary material for: Caffeine Use in Huntington’s Disease: A Single Center Survey
Source: Tremor Other Hyperkinet Mov (N Y). 2024 Oct 18;14:52. doi: 10.5334/tohm.945 (PMC11488189; doi:10.5334/tohm.945)
Supplement: Appendix A. — Study Survey. [file tohm-14-1-945-s1.pdf]

**Caffeine in Huntington's Disease (HD)**
Study Participant ID: ____________
Today's Date: __________________

**Did family/care partner(s) assist or give input during the survey?**

☐ Yes
☐ No

If so, what is the relationship? Please select all that apply.

☐ Family
☐ Friend
☐ Care partner (not biological or legal family)

**Background:**

1. **Age Range (yrs):** 18-24 25-34 35-44 45-54 55-64 65 and older
2. **Sex assigned at birth:** ☐ Male ☐ Female
3. **Race (check all that apply):**
   ☐ White/Caucasian
   ☐ Black or African American
   ☐ American Indian or Alaska Native
   ☐ Asian
   ☐ Native Hawaiian or Other Pacific Islander
   ☐ Other: ___________________
4. **Ethnicity:** ☐ Hispanic/Latino ☐ Not Hispanic/Latino
5. Have you had motor (physical, movement-related) HD symptoms? ☐ Yes ☐ No
   What year did they first start? ________
   Are you on medications to treat motor symptoms related to HD? ☐ Yes ☐ No
6. Have you had psychiatric (depression, anxiety, agitation) HD symptoms? ☐ Yes ☐ No
   What year did they first start? ________
   Are you on medications to treat mood symptoms related to HD? ☐ Yes ☐ No

Total Functional Capacity

Please select the most applicable answer to the following.

1. If I had to continue or return to my most recent job/occupation, I would:
   - ☐ be able to work **normally** (successfully without any assistance)
   - ☐ be able to work at **reduced capacity** for usual job
   - ☐ be able to do **marginal work only**
   - ☐ **not be able** to work my usual job at all

2. If I had to manage my finances (monthly bills, taxes, etc.), I would:
   - ☐ be able to manage the finances **normally and independently**
   - ☐ be able to manage the finances with **minor assistance**
   - ☐ be able to manage the finances with **major assistance**
   - ☐ **not be able** to manage the finances at all.

3. If I had to do domestic chores (washing dishes, cleaning, laundry, etc.), I would:
   - ☐ be able to do the chores **normally and independently**
   - ☐ be able to do the chores **with some difficulty (impaired)**
   - ☐ **not be able** to do the chores at all.

4. For my activities of daily living (bathing, dressing, feeding self), I am:
   - ☐ able to do them **normally and independently**
   - ☐ able to do them with **minimal help (minimal impairment)**
   - ☐ able to do them with **a lot of help**
   - ☐ **totally dependent on others** for these tasks

5. How would you describe your home living setting?
   - ☐ Home (mostly independent)
   - ☐ Home with caregiver(s)
   - ☐ Full-time nursing care

**Caffeine Intake Questionnaire**

1. During a usual day, how many of the following caffeinated beverages or products do you consume on average **daily**? Please specify the count of each per day.

| | **Small (8oz)** | **Medium (12 oz)** | **Large (16 oz)** | **Extra Large (20 oz or more)** |
|---|---|---|---|---|
| **Coffee** (please specify)<br><br>☐ Decaffeinated<br>☐ Normal Caffeinated<br>☐ Extra Caffeinated | | | | |
| **Black or caffeinated tea** | | | | |
| **Energy Drink** | | | | |
| **Soda/Pop** (please specify)<br><br>☐ Caffeinated<br>☐ Caffeine-free | | | | |
| **Caffeine Tablets** | | | | |
| **Other: _______________** | | | | |

**Please specify:**
Ex: “2 large mugs of black coffee, 1 Starbucks Venti (20 oz) cappuccino, and 1 shot of expresso daily”

______________________________________________

______________________________________________

______________________________________________

______________________________________________

2. Have you ever felt that you should cut down on your caffeine intake?
   - ☐ Yes
   - ☐ No

3. Have people in your life recommended or suggested that you cut down on your caffeine intake?
   - ☐ Yes
   - ☐ No

4. Have <u>you</u> noticed that caffeine affects your HD symptoms?
   - ☐ Yes
   - ☐ No

   For each of the following symptoms, please specify if caffeine makes them 1 = Worse, 2 = No change, 3 = Better, 0 = Not Sure

| | Worse | No Change | Better | Not Sure |
|---|---|---|---|---|
| Chorea | 1 | 2 | 3 | 0 |
| Dystonia | 1 | 2 | 3 | 0 |
| Tremor | 1 | 2 | 3 | 0 |
| Anxiety | 1 | 2 | 3 | 0 |
| Depression | 1 | 2 | 3 | 0 |
| Gait | 1 | 2 | 3 | 0 |
| Irritability or anger | 1 | 2 | 3 | 0 |
| Other: _______________ | 1 | 2 | 3 | 0 |

5. Have <u>family, loved ones, or care partners</u> noticed that caffeine affects your HD symptoms?
   - ☐ Yes
   - ☐ No

   For each of the following symptoms, please specify if caffeine makes them 1 = Worse, 2 = No change, 3 = Better, 0 = Not Sure

| | Worse | No Change | Better | Not Sure |
|---|---|---|---|---|
| Chorea | 1 | 2 | 3 | 0 |
| Dystonia | 1 | 2 | 3 | 0 |
| Tremor | 1 | 2 | 3 | 0 |
| Anxiety | 1 | 2 | 3 | 0 |
| Depression | 1 | 2 | 3 | 0 |
| Gait Stability/Balance | 1 | 2 | 3 | 0 |
| Irritability or anger | 1 | 2 | 3 | 0 |
| Other: _______________ | 1 | 2 | 3 | 0 |

   This question was answered by: (Please select one)
   - ☐ Participant with HD
   - ☐ Family/Friend/Care Partner(s)

6. For each one of the following statements below, select the response that best characterizes how you feel about the statement, where 1 = Strongly Disagree, 2 = Disagree, 3 = Neither Agree nor Disagree, 4 = Agree, 5 = Strongly Agree, 0 = Not Sure

**I drink caffeinated beverages or take caffeine supplements, because…**

| | Strongly Disagree | Disagree | Neither Agree nor Disagree | Agree | Strongly Agree | Not Sure |
|---|---|---|---|---|---|---|
| I like the taste. | 1 | 2 | 3 | 4 | 5 | 0 |
| It makes me feel less sleepy. | 1 | 2 | 3 | 4 | 5 | 0 |
| It gives me extra energy. | 1 | 2 | 3 | 4 | 5 | 0 |
| It helps me think clearer and concentrate. | 1 | 2 | 3 | 4 | 5 | 0 |
| Other people around me do it, too | 1 | 2 | 3 | 4 | 5 | 0 |
| Caffeine is good for my health. | 1 | 2 | 3 | 4 | 5 | 0 |
| My mood is better with it | 1 | 2 | 3 | 4 | 5 | 0 |
| I get a headache if I don't | 1 | 2 | 3 | 4 | 5 | 0 |
| I don't know why I like it | 1 | 2 | 3 | 4 | 5 | 0 |

Other comments:
